# A short review of current knowledge regarding long-term treatment of Graves’ disease with antithyroid drugs

**DOI:** 10.1007/s42000-024-00618-y

**Published:** 2024-12-05

**Authors:** Ilaria Giordani, Gerasimos P. Sykiotis

**Affiliations:** Service of Endocrinology, Diabetology and Metabolism, Lausanne University Hospital and University of Lausanne, Lausanne, Switzerland

**Keywords:** Graves’ disease, Hyperthyroidism, Long-term treatment, Antithyroid drugs

## Abstract

Graves’ disease is the most common form of hyperthyroidism, especially in younger people. Current European guidelines recommend antithyroid drugs as initial treatment for a period limited to 12–18 months. Definitive treatment such as surgery or radioactive iodine is proposed in the case of contraindication to antithyroid drugs or in the case of recurrence after medical treatment. However, more recent studies show that long-term antithyroid treatment is associated with reduced risk of recurrence without an increase in adverse effects. Such data support the option of long-term treatment of Graves’ disease with antithyroid drugs and suggest the necessity for a change to long-standing practices in the field. Herein, after reviewing some general knowledge on Graves’ disease treatment, we discuss the evidence regarding long-term treatment of Graves’ disease with antithyroid drugs for endocrinologists, internists, and other specialists involved in the management of these patients. We consider the main studies in the field, outline their respective strengths and limitations, and, finally, present our opinion on when, in the light of this new evidence, endocrinologists should consider long-term treatment with antithyroid drugs.

## Introduction

Graves’ disease is an autoimmune thyroid disorder that manifests as hyperthyroidism secondary to the production of autoantibodies that bind and activate the TSH (thyroid-stimulating hormone) receptor on the surface of thyroid follicular cells. As in the case of several other autoimmune diseases, the cause is unclear and appears to be related to a combination of genetic and environmental factors [1, 2]. Patients usually display symptoms such as weight loss, palpitations, tremor, diarrhea, nervousness, insomnia, heat intolerance, etc. The clinical examination typically reveals tachycardia, hand tremor, hyperreflexia, and a diffuse goiter with a possible thyroid bruit. There are also possible extrathyroidal manifestations, including thyroid eye disease (TED) and thyroid acropachy (also known as thyroid dermatopathy) [3, 4]. The clinical presentation may vary depending on the cause and on the degree of hyperthyroidism and on the age and sex of the patient. Older adults can present with fatigue, apathy, and depression without the classical adrenergic symptoms (e.g., apathetic thyrotoxicosis) [5]. Diagnosis is based on the presence of anti-TSH receptor antibodies (TRAb) associated with hyperthyroidism. Ultrasound typically shows a diffuse goiter with increased vascularity and thyroid scintigraphy usually shows diffuse thyroid uptake, although the latter is often not necessary to confirm the diagnosis.

## Graves’ disease treatment

Three therapeutic options are currently proposed, namely, antithyroid drugs (ATD), radioactive iodine (RAI) or total thyroidectomy. ATD are the most widely used initial treatment in Europe, whereas RAI has been historically the treatment of choice in the USA.

However, recent data have documented an increased preference of ATD compared to RAI in the USA, as shown by two population studies [6, 7]. This trend has also been confirmed by a recent global online survey conducted in 2023 showing ATD as the preferred therapy for uncomplicated Graves’ disease by 91.5% of participants [8, 9].

Treatment with ATD can restore euthyroidism and facilitate durable remission of the disease in many patients; in contrast, total thyroidectomy tends to cause hypothyroidism, followed by levothyroxine substitution. This is often also the case for RAI, depending on the administered activity and local practice.

### ATD

Two types of ATD are in clinical use, namely, imidazoles (carbimazole and methimazole) and thiouracils (propylthiouracil and benzylthyouracil). They inhibit the synthesis of thyroid hormones by blocking the action of the enzyme thyroperoxidase. Propylthiouracil (PTU) also inhibits monodeiodinase type 1, thereby decreasing the peripheral conversion of thyroxine (T4) to thyronine (T3).

In countries where methimazole (MMI) is not available, the most widely used antithyroid drug is carbimazole (CBZ), a precursor drug that is rapidly converted to MMI (10 mg of CBZ are roughly equivalent to 6 mg of MMI).

The mechanism by which ATDs contribute to autoimmune remission is not fully understood, but the main hypothesis is the presence of an immunomodulatory effect [10, 11]. One explanation is an antioxidant effect mediated by MMI via the inhibition of the INF-gamma pathway and the elimination of hydrogen peroxide [12]. Moreover, MMI inhibits lymphocyte proliferation in vitro [13]. It has also been demonstrated that patients with Graves’ disease treated with MMI had lower serum levels of cytokines compared to untreated patients [14].

Beyond side effects (which are discussed later), the main disadvantage of treatment with ATD is the risk of recurrence after discontinuation of treatment, estimated at 30–70%, depending on the studies [15]. Factors associated with a higher probability of recurrence include male sex, age < 40 years, tobacco consumption, severe hyperthyroidism, high TRAb titer, increased thyroid volume and the presence of TED [16, 17], but each single risk factor has small predictive power. A 2017 metanalysis including 31 trials for pretreatment risk of relapse reported that TED, smoking, thyroid volume, higher TRAb and higher free thyroid hormone concentrations were associated with a higher risk of relapse. Contrary to other studies, male sex was not associated with higher risk of relapse [17].

A predictive model based on clinical factors (GREAT score) or on a combination of clinical and genetic factors (GREAT + score) has been proposed [18]. The scores are based on pre-treatment patient characteristics such as younger age, higher free T4 concentration, higher TRAb titer, goiter size, and presence of specific HLA polymorphisms or PTPN22 polymorphisms. The higher the score, the higher the risk of recurrence after discontinuation of ATD. The GREAT score has been validated in three independent cohorts of patients [19–21].

Regarding the risk of recurrence at the time of ATD withdrawal, predictive factors have been identified in various studies; the most consistently reported ones include young age, male sex, smoking, higher TRAb values, higher maintenance dose of MMI and higher thyroid volumes [15, 22–30].

A study on a cohort of 549 patients with a long follow up (8.6–36.4 years) showed that remission was more frequent (88.9%) in the group of patients with negativization of TRAb than in patients with fluctuating titers (37.2%) or patients with persistent positive TRAb (19.8%) [31].

### Surgical treatment

Surgical treatment involves total thyroidectomy, which is currently preferred to subtotal thyroidectomy given the risks of recurrence with the latter (8% compared to almost 0% for a total thyroidectomy) [32], as well as the high probability of developing hypothyroidism (approximately 50% of patients treated with subtotal thyroidectomy). Following thyroidectomy, TRAb decrease rapidly during the first 9 postoperative months; nevertheless, they remain detectable in 18% of patients even after 3 years [33]. In patients with TED, surgical management does not influence the clinical outcomes of orbitopathy [34].

### RAI

Therapy with RAI consists of the administration of an iodine 131 capsule with an activity that is either empirically determined or calculated according to the volume of the gland and the rate of iodine 123 uptake (this latter practice, which is mandated by the health authorities in some countries, requires a prior diagnostic examination). With varying percentages depending on the study, most patients progress to hypothyroidism (> 80% of patients after 16 weeks [35]), a minority remain euthyroid, and some require a second (or, even more rarely, third) RAI treatment due to persistent hyperthyroidism (up to 14% of patients [36]). Three years after RAI, up to 60% of patients have detectable TRAb [33]. A few studies have observed that persistence of high levels of TRAb is associated with risk of recurrence and TED [37, 38]. Consideration should also be given to the possibility of deterioration or development of TED following RAI. The incidence or deterioration of TED in patients treated with RAI has been shown to be significantly higher than in patients treated with ATD [39, 40]. Thus, the presence of moderate-to-severe or sight-threatening TED is generally considered as a contraindication for this treatment.

## Current recommendations

**Fig. 1:**
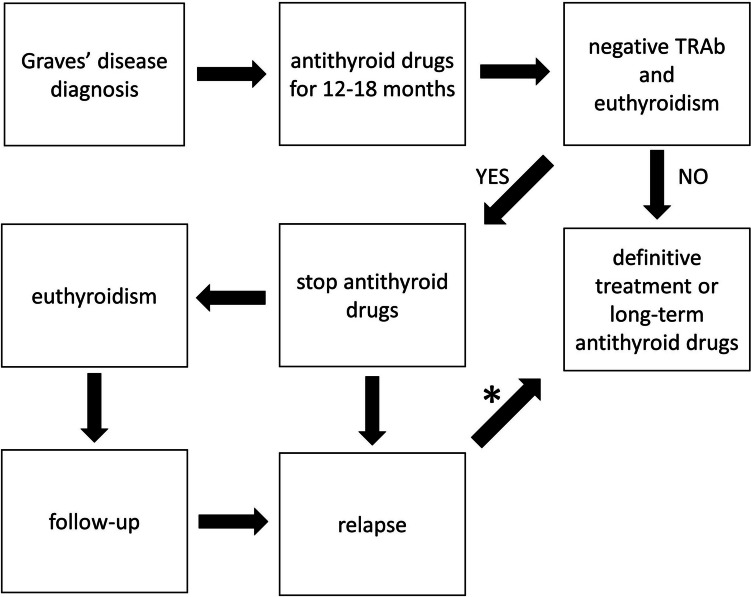
Proposed algorithm for the treatment of hyperthyroidism. *Studies on the long-term use of ATD have only been done in patients with Graves’ disease during their first course of ATD. There are no data so far on the long-term use of ATD after a relapse

The 2018 European guidelines (Fig. 1) recommend treatment with ATD for 12–18 months. Provided that the TSH level is within the normal range and that TRAb are negative, treatment is discontinued. In the case of persistently positive TRAb, treatment beyond 12–18 months might be considered as an alternative to radioactive iodine treatment or surgery [41].

These recommendations are based on several studies from the 1990s, showing that there is no benefit in terms of relapse rate in continuing treatment with ATD beyond 18 months [42, 43]. However, no study addressed a treatment duration of more than 42 months.

Definitive treatment (surgery or RAI) is indicated if TRAb remain positive after treatment with ATD or in the case of recurrence after ATD withdrawal. In the latter case, a second course of ATD may be proposed. There are no data so far on the long-term use of ATD after a relapse. A few studies have shown a modest decrease in recurrence rates with a second, third, or fourth standard course, respectively [44, 45].

### Pregnancy and preconception

In women of childbearing age, the discussion around therapeutic options should take into consideration the timing of family planning, the presence of a goiter, the possible lifelong need for levothyroxine therapy, and the contraindications and adverse events of each option [46]. Conception is contraindicated for at least 6 months following radioiodine treatment, which implies that RAI would not be an option for women planning a pregnancy in this period. Conception is also contraindicated in men for at least 6 months following the administration of radioiodine. In the case of treatment with ATD, it is important to closely monitor thyroid function in the preconception period, and it is common practice to switch from MMI to the less teratogenic PTU during the first trimester of pregnancy. In women diagnosed with Graves’ disease during pregnancy, the first line treatment is ATD with close fetal and maternal monitoring. Thyroidectomy should be considered in the case of severe allergy or other contraindications to ATD or in the event of persistence of thyrotoxicosis despite ATD treatment [46]. In patients with pre-existing Graves’ disease and a newly confirmed pregnancy, if hyperthyroidism is well controlled on a low dose of ATD, stopping the treatment and closely monitoring the patient is possible since the immunotolerant state of pregnancy usually allows the maintenance of euthyroidism. Moreover, should a relapse occur, this would generally be several weeks after the withdrawal of ATD and thus probably after the period when the patient is most at risk for teratogenic effects (gestational weeks 6–10).

## Long-term antithyroid therapy

Currently, accumulating data support the option of a long-term (and in some cases lifelong) treatment with ATD. We discuss the main studies in the field, outline their respective strengths and limitations, and, finally, present our opinion on when, in the light of this new evidence, endocrinologists should consider long-term treatment with antithyroid drugs.

Advantages of long-term antithyroid therapy include the prevention of relapse and the potential restoration of euthyroidism. Compared to the conventional treatment, long-term antithyroid treatment has been associated with a higher chance of Graves’ disease remission. It also allows for preservation of thyroid function, as opposed to RAI and thyroidectomy. Additionally, this treatment does not involve radiation exposure or surgical risks and hospitalization. In terms of disadvantages, side effects associated with long-term ATD are rare but might be severe. Moreover, the patient should be compliant with long-term treatment and follow-up, although this may not differ substantially from the lifelong follow-up of a patient with substituted hypothyroidism. Finally, there are limited data on the safety and efficacy of long-term ATD therapy.

We conducted a review of the literature on long-term ATD treatment; we excluded case reports, pediatric cohorts, studies exclusively on toxic goiter, cohorts with less than 2 years of median ATD treatment, and studies in which the remission/relapse rate was not an outcome. The results of the most relevant studies on long-term treatment of Graves’ hyperthyroidism are summarized in Table 1.

**Table 1:** Studies on long-term ATD treatment in adult patients with Graves’ disease

Study	Type of the study	Population	Follow-up	Outcome	Side effects of ATD treatment	Limitations	Comments
Shizume 1978 [47]	Retrospective	14 patients with recurrence of Graves’ disease who received ATD treatment for 8 to 21 years	8–21 years of therapy, only 3 patients discontinued the treatment during the observation	Stable thyroid function with low dose ATD treatment	No side effects observed during 8 to 21 years of therapy	No data on remission rate as patients were still being treated	
Maugendre et al. 1999 [48]	Prospective randomized trial	62 patients treated with carbimazole for 18 months vs. 72 patients treated for 42 months	2 years after treatment discontinuation	No difference in the relapse rate (36% vs. 29%, NS)	5 patients developed adverse effects (not otherwise specified)	Treatment long-term 42 months maximum	The percentage of patients with TRAb was significantly lower in the 42-month treatment group (18% vs. 42%, p 0.004) at treatment withdrawal, but the percentage of TRAb-positive patients did not significantly decrease between 18 and 42 months in this group (27% vs. 18%, NS).
Mazza et al. 2008 [49]	Retrospective	384 patients newly diagnosed with Graves’ disease, treated with MMI for more or less 15 months – two age groups, > and < 35 years	Minimum 24 months	No difference in relapse in patients < 35 years between the two groups. Risk of relapse increased in patients > 35 years in which treatment was discontinued before 15 months	No case of agranulocytosis, one case of doubtful hepatotoxicity which resolved without discontinuation; 8 cases of urticaria	Exclusion of patients who needed high doses of MMI	
Morales García et al. 2008 [50]	Prospective	53 patients on long term low dose ATD therapy (group M) vs. 31 patients who stopped ATD (group R)	Mean follow-up 31.6 months for group R and 49.4 months for group M	Relapse was observed in 12/53 patients in group M (22.64%) and in 24/31 patients in group R (77.42%)	Not described	Retrospective (group M)	
Elbers et al. 2011 [51]	Retrospective	73 patients with Graves’ disease and TED received block-and-replace ATD treatment for a median of 41 months	Mean follow-up 57 months (range: 12–170)	Recurrence rate of hyperthyroidism of about 37%	Not described		No case of worsening of ophthalmopathy after discontinuation of ATD nor in patients treated with radioactive iodine
Laurberg et al. 2011 [52]	Retrospective	108 patients with Graves’ disease and TED on partial block and replace regimen	Mean follow up 80 months	Only four (3.7%) developed episodes of hyperthyroidism during stable therapy, and 94% had serum TSH within 0.1–4.0 mU/L at the last observation	7 patients with suspected cutaneous reaction to MMI switched to PTU One patient with slightly elevated liver enzymes one patient with moderate arthralgia One patient with vasculitis after 6 years of PTU therapy	Retrospective	Nearly all patients received immunosuppressive treatment for ophthalmopathy
Konishi et al. 2011 [53]	Retrospective	107 patients with Graves’ disease who concluded ATD therapy	Minimum 2 years	The percentage of patients in remission was 86.9% at 6 months, 73.8% at 1 year, and 68.2% at 2 years after ATD discontinuation	Nor reported	Retrospective	TRAb levels at the time of ATD withdrawal in the relapse group were significantly higher than those in the remission group
Villagelin et al. 2015 [54]	Retrospective	124 out of 238 patients with relapse of Graves’ after 12–24 months conventional ATD regimen (vs. RAI)	80.8 ± 35.3 months for the RAI group, and 71.3 ± 40.3 months for the low-dose MMI group	Higher frequency of euthyroid status in the low-dose MMI dose group versus the RAI group	No reports of major adverse effects of MMI	Retrospective Selection bias	GO evolution more favorable in the low-dose MMI group than it was in the RAI group Trend to body weight increase in the RAI group No differences in QoL
Azizi et al. 2019 [55]	Randomized clinical trial	302 patients with untreated first episodes of Graves’ randomized to continue ATD for 60–120 months or to discontinue after 18–24 months	48 months after MMI discontinuation	Hyperthyroidism recurrence within 48 months in 15% of long-term patients versus 53% of conventional group patients	14 patients with cutaneous reactions and two liver enzyme elevations during the first 18 months of treatment; no adverse reaction after the first 18 months	Not double-blind West Asian ethnicity only TRAb levels measured only before initiation and at withdrawal	Lower TRAb levels were associated with a higher rate of sustained remission
Park et al. 2021 [56]	Retrospective	908 patients with first diagnosis of Graves’ disease	Mean follow up 79.9 ± 33.7 months	Relapse rate according to ATD treatment duration was 42.4% at 1 year, 38.5% at 2 years, 33.8% at 3 years, 31.7% at 4 years, 30.2% at 5 years, 27.8% at 6 years, and 19.1% at more than 6 years	26 patients (2.9%) experienced minor side effects (pruritus or rash), without stopping ATD treatment	Retrospective No TRAb dosage before ATD discontinuation Iodine-replete area	
Azizi et al. 2021 [30]	Clinical trial	59 patients with Graves’ disease already on long-term MMI, 32 (54%) decided to discontinue and 27 (46%) decided to continue)	6 additional years	In the group who continued the treatment, no suppressed TSH. In the group who discontinued MMI, 6 relapses (19%) and 1 patient lost to follow-up.	No adverse reaction during additional years of treatment	Not randomized	Mean daily dose of MMI to maintain TSH in the reference range decreased gradually and reached 2.8 ± 1.7 mg by 24 years of MMI treatment.
Lertwattanarak et al. 2022 [57]	Randomized clinical trial	184 patients with first diagnosis of Graves’ disease, randomized to discontinue MMI or continue low-dose MMI	36 months	Lower recurrence in the low-dose MMI group (1.2% vs. 11.2%, 6.8% vs. 18.4%, 11.0% vs. 27.2%, 11.0% vs. 35.0%, and 11.0% vs. 41.2% at 6, 12, 18, 24, and 36 months)	Neither minor nor major adverse effects of low-dose MMI therapy	Lack of TRAb results at first diagnosis and at randomization	
Saadat et al. 2022 [58]	Randomized clinical trial	64 patients with recurrence of hyperthyroidism (either toxic nodular or toxic diffuse goiter) after RAI, randomly assigned to long-term MMI or RAI	60 months	Shorter time to euthyroidism in MMI group (3.5 vs. 9.5 months) and higher time spent in euthyroidism in MMI group (95.2% vs. 77.7%)	No major adverse events (agranulocytosis, hepatic necrosis or autoimmune disease), minor side effects only in the first 4 months not otherwise specified	Not double-blind Patients with nodular or toxic diffuse goiter	
Azizi et al. 2024 [59]	Randomized clinical trial	258 patients with untreated first episode of Graves’ disease, randomized to conventional duration (18–24 months) versus long duration (60–120 months) MMI therapy	84 months	17% vs. 56% of recurrence in patients who received long-term methimazole treatment	No major event Minor side effects (skin reaction, elevated liver enzymes) in 5.3% of patients in the first 2 months. No AEs after month 3.	Not double-blind 44 patients were excluded before randomization	

A 2015 study demonstrated the efficacy of MMI treatment in patients with recurrent Graves’ disease after a first course of ATD. Treatment with low-dose MMI (2.5–7.5 mg/day) for 60 months was associated with higher rates of euthyroidism compared to treatment with RAI, as well as with better results in terms of TED [54].

A retrospective study showed that the duration of ATD is directly correlated with remission rates after stopping treatment, with a recurrence rate of 19% for patients treated for > 6 years [56]. This study is consistent with the results of a 2017 metanalysis that demonstrated a remission rate of 16% for each year of treatment [60].

Most of the data on long-term ATD come from retrospective studies like the aforementioned ones. The retrospective analysis might be associated with a selection bias due to the nature of the studies. For example, patients who received a definitive treatment (either RAI or surgery) might have had more severe hyperthyroidism than patients selected for long-term ATD.

One prospective study confirmed the benefit of continuing treatment with ATD beyond the recommended duration in patients with Graves’ disease in euthyroidism on low-dose MMI (2.5–5 mg/day) [57]. In this study, patients who continued low-dose MMI had a 3.8 times lower risk of recurrence compared to patients who stopped the drug after achieving euthyroidism.

A recent randomized trial showed a lower risk of recurrence in patients treated with long-term MMI treatment (17% of recurrence in patients treated for 60–120 months versus 56% in patients treated for a median of 18 months) [61].

The cohort with the most extended follow-up under MMI treatment, described in 2021, had an average treatment duration of 14.2 years [30]. Of the 32 patients who discontinued treatment, only 19% had recurrence of hyperthyroidism. The group of 27 patients who continued treatment up to 24 years of follow-up had unsuppressed TSH values, MMI mean daily doses of 2.8 mg/day (+/- 1.7 mg), negative TRAb and no reported adverse effects after the first 24 months of treatment. Medical treatment also presents risks: apart from minor adverse effects (skin rashes, arthralgia), there is a risk of severe and potentially lethal complications, such as agranulocytosis or drug-induced hepatitis, vasculitis, or pancreatitis. Adverse effects usually occur during the first 3–6 months of treatment and appear to be associated with higher doses [62].

Confirming these observations, a 2019 literature review analyzed the frequency of adverse effects of ATD in patients on long-term treatment with MMI (1660 patients, mean treatment duration of 2.3–14.2 years) [63]. The authors concluded that minor adverse effects were quite frequent (2–36% depending on the study), the most common being skin reactions, abnormal liver function test, leukocytopenia and arthralgia. Only 14 patients experienced severe adverse effects, including agranulocytosis (*n* = 7), hepatitis (*n* = 5), glomerulonephritis (*n* = 1) and vasculitis (*n* = 1). Moreover, only five patients presented adverse effects after the first year of treatment.

A recent study on the long-term treatment of Graves’ disease with MMI confirms the safety of the treatment during a 48-month follow-up. About 10% of patients experienced adverse effects (mostly skin reactions), with 75% of them manifesting within the first 6 months of treatment. No adverse effects were described in patients who continued treatment with 5 mg/day of MMI after 24 months [64].

Regarding pregnancy, there are no data specifically on long-term ATD treatment in women of childbearing age. However, if pregnancy is confirmed in a patient with Graves’ disease on long-term ATD treatment, thanks to the immunotolerant state of pregnancy, in most cases it is possible to withhold the low-dose treatment while closely following the patient (and, if indicated, the fetus).

### Long-term ATD in the setting of multinodular goiter with or without Graves’ disease

In the setting of nonfunctioning nodules coexisting with Graves’ disease, the presence of large thyroid nodules, compressive symptoms or suspicion of malignancy should favor surgical treatment over the other options, depending also on patient preferences, age and comorbidities, and access to a high-volume surgeon [65].

For toxic multinodular goiter, definitive treatment (thyroidectomy or RAI) is usually preferred to treatment with ATD because hyperthyroidism relapses upon discontinuation of antithyroid drug therapy in 95% of patients [66]. There are very few studies on the long-term medical treatment of hyperthyroidism in the context of toxic multinodular goiter. A randomized, controlled study published in 2019 compared the efficacy and safety of long-term MMI treatment to RAI in patients with toxic multinodular goiter. The authors demonstrated that long-term treatment with MMI achieves euthyroidism in 89% of patients, with an average dose of 5 mg/day from the second year of treatment [67]. As with Graves’ disease, the rare adverse effects occurred during the first 3 months of treatment, and no patient developed an adverse event between the 4th and 100th month of follow-up.

## Conclusion

The long-term medical treatment of hyperthyroidism with ATD is effective and with limited side effects, and it can be considered as a perfectly valid alternative in the treatment of Graves’ disease, and potentially also of toxic multinodular goiter, even though additional evidence from randomized trial would be welcome for both indications.

As is the case for the choice of initial treatment for Graves’ hyperthyroidism, the consideration of long-term ATD treatment later during the course of the disease depends on patient characteristics and comorbidities as well as on patient preference. Advantages and disadvantages of the different treatment modalities should be discussed in detail with the patient, with particular consideration for women of childbearing age. As a general principle, because adverse events of ATD after the first year of treatment are rare and dose-related, long-term low-dose treatment can be considered safe.

Therefore, in our opinion, continuous ATD treatment can be proposed as an alternative to patients in whom definitive treatment would otherwise be indicated in order to avoid permanent hypothyroidism and to diminish the risk of subsequent relapses. It can also be considered in patients who want to delay definitive treatment in a particular moment of their life fearing a relapse. Stressful events, in particular, have been associated with relapse of hyperthyroidism in patients with Graves’ disease [68]. Patients who refuse definitive treatment or have contraindications to it are also obvious candidates. In patients with persistent disease or with multiple relapses who do not desire ablation, lifelong treatment can be discussed. These considerations are generally consistent with the approach recently formulated by Azizi et al. [69].

Because no guidelines exist to guide the follow-up of patients on long-term ATD or to decide whether and when to stop ATD, it is important to identify those at higher risk of relapse after ATD withdrawal. These patients could benefit from a prolonged (> 60 months) course of treatment, as this duration has been shown to provide a 4-year remission rate of 85% [70].

A recently proposed algorithm by Azizi et al. [71] suggests an assessment of TRAb after 18 months of ATD treatment. If TRAb values are undetectable (< 0.9 U/L), withdrawal should be considered. Otherwise, treatment should be continued for 5 years with biannual monitoring of TSH and free T4 to adjust the ATD dose. If after 5–6 years of ATD treatment TRAb are < 0.9 U/L, then discontinuation should be discussed. The same authors propose a predictive risk score for relapse at the time of discontinuation [59]. This score (ranging from 0 to 14) is based on duration of treatment (short term = 6 points), sex (male = 1 point), age (> 50 years = 1 point), fT4 (> 17 pmol/L = 3 points), T3 (> 120 ng/dl = 1 point), TSH (< 1 mU/L = 1 point), TRAb (> 1.75 U/mL = 3 points), and goiter (presence = 2 points). A lower score (< 5) is associated with a lower risk of recurrence (7% vs. 70% of recurrence with a score of 10–14).

In conclusion, we encourage fellow endocrinologists to consider this approach in eligible patients, and we propose that further testing of the respective practices in international settings is warranted, including studies on the long-term use of ATD have after GD relapse.

## Data Availability

The authors confirm that the data supporting the findings of this study are available within the article.
